# Efficacy of specific immunotherapy of abdominal sepsis

**DOI:** 10.1186/cc12907

**Published:** 2013-11-05

**Authors:** Olexandr Butyrsky, Iryna Butyrsky

**Affiliations:** 1 Department of Surgical Diseases, Crimean State Medical University, Simferopol, Ukraine; 2 Department of Hygiene, Crimean State Medical University, Simferopol, Ukraine

## Background

According to the 2004 WHO Annual Report, abdominal sepsis (AS) is one of the most dangerous diseases of the 21st century. But the issue of its treatment including immunotherapy is very far from being completely solved. Our aim was the demonstration of specific immunotherapy efficacy in AS.

## Materials and methods

We investigated activity of immunogenesis (production of specific antibodies) in AS in 50 patients under impact of hyperimmune plasma infusion obtained from the donors who recently endured acute inflammatory abdominal diseases. The control group was made up of 10 healthy people.

## Results

Our investigations demonstrated that the donors' titer of specific antibodies is evidently more according to indexes of anti-*Escherichia coli*, anti-*Pseudomonas aeruginosa*, anti-*Staphylococcus aureus*, anti-bacteroids, anti-peptococci immunity. Introducing hyperimmune plasma obtained from such donors evidently increases specific antibody titer in AS patients (Table 1).

**Table 1:** Dynamics of specific antibody titer in AS patients after hyperimmune plasma introduction

	Before introducing, units/l	After introducing, units/l
Anti-*E. coli *(*n *= 10)	3.27 ± 0.23	4.15 ± 0.31*
Anti-*P. aeruginosa *(*n *= 10)	3.15 ± 0.41	3.94 ± 0.21*
Anti-*S. aureus *(*n *= 10)	3.06 ± 0.31	4.02 ± 0.45*
Anti-bacteroids (*n *= 10)	3.56 ± 0.28	4.36 ± 0.35*
Anti-peptococci (*n *= 10)	3.76 ± 0.39	4.51 ± 0.32*

*Parameters change evidently (*P *< 0.05).

Our results demonstrated that the titer of specific antibodies to AS main agents in the dead patients was evidently lower in comparison with the control group and in recovered people. This trend is observed 7 to 10 days after surgery. For example, at admission the titer of anti-*S. aureus *antibodies in dead patients was 11% lower in comparison with the control group, and the titer of anti-*E. coli *antibodies 19% lower in comparison with control group and 32% lower in comparison with recovered patients; 7 to 10 days after surgery in dead patients in comparison with recovered patients, the titer of anti-*S. aureus *antibodies was lower by 22%, and the titer of anti-*E. coli *antibodies by 47%.

## Conclusions

Introducing hyperimmune plasma (specific immunotherapy) increases titer of specific antibodies, and increased concentration of specific antibodies improves forecast of survival in AS.

